# Diagnostic value of procalcitonin and hemocyte parameters in neonates with bloodstream infection: Role of activated hemocyte‐related genes

**DOI:** 10.1002/pdi3.56

**Published:** 2024-05-23

**Authors:** Yiyi Tao, Qian Li, Huidi Peng, Ningshu Huang

**Affiliations:** ^1^ Department of Clinical Laboratory Chongqing Key Laboratory of Pediatrics Ministry of Education Key Laboratory of Child Development and Disorders National Clinical Research Center for Child Health and Disorders China International Science and Technology Cooperation Base of Child Development and Critical Disorders Children's Hospital of Chongqing Medical University Chongqing China; ^2^ Department of Clinical Laboratory Women and Children's Hospital of Chongqing Medical University Chongqing China; ^3^ Department of Clinical Laboratory Chongqing Health Center for Women and Children Chongqing China; ^4^ Department of Clinical Laboratory The Third People's Hospital of Longgang District Shenzhen Shenzhen China

**Keywords:** bloodstream infection, hemocyte parameters, hemocytes activation, neonates, procalcitonin

## Abstract

This study aimed to evaluate the diagnostic potential of hemocyte parameters and procalcitonin (PCT) in detecting bloodstream infections (BSI) in neonates and explore the contribution of hemocyte activation‐related genes to pediatric sepsis through bioinformatics analysis. A cohort of 419 neonates, categorized as BSI (positive blood culture) and control, underwent comparative analysis of PCT and hemocyte parameters. A predictive model for neonatal BSI was established, demonstrating an impressive area under the receiver ROC curve of 0.968 with remarkable sensitivity (92%) and specificity (87.3%). Hemocyte parameters, including lymphocyte and neutrophil percentages, platelet distribution width (PDW), platelet to lymphocyte ratio (PLR), and PCT, emerged as independent predictors of neonatal BSI. Furthermore, bioinformatics analysis utilizing Gene Expression Omnibus (GEO) datasets yielded significant insights. Differential gene expression (DEGs), gene ontology (GO), pathway enrichment, gene set enrichment analysis (GSEA), and protein–protein interaction (PPI) networks were explored. The differentially expressed genes and hub genes were notably enriched in the activation of neutrophils, lymphocytes, and platelets. Notably, elevated expression levels of SPI1, TYROBP, and FCER1G were observed in pediatric sepsis or septic shock, with positive correlations between SPI1, FCER1G, and TYROBP. In summary, the combination of lymphocyte, PDW, PLR, and PCT effectively diagnosed neonatal BSI. Bioinformatics analysis underscored the pivotal role of activated hemocytes in diagnosing pediatric sepsis, with SPI1, TYROBP, and FCER1G co‐expression influencing the disease's pathophysiology by modulating neutrophil and platelet activity.

## Graphical abstract

Our model, incorporating accessible biomarkers (PCT, LY%, NE%, PDW, and PLR), demonstrated enhanced neonatal BSI diagnosis. Our study unveiled the pivotal role of the SPI‐TYROBP‐FCER1G co‐expression network in pediatric sepsis immune response, impacting neutrophil and platelet activation, potentially indicating novel biomarkers.

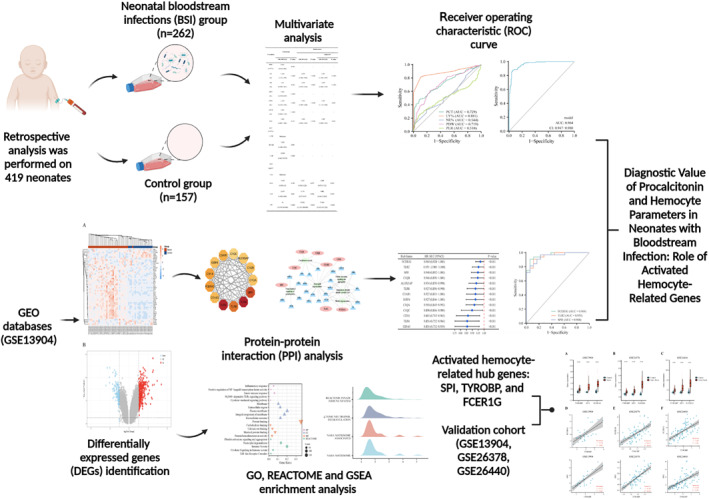

## 1 INTRODUCTION

The susceptibility of neonates to bloodstream infections (BSI) is increased due to their immature immune system, resulting in the development of neonatal sepsis. Recent studies demonstrated that sepsis and BSI remain major causes of pediatric morbidity and 10%–20% mortality worldwide.^1^, ^2^ A global cross‐sectional study has indicated that the prevalence of sepsis among children in pediatric intensive care units was 8.2%, and there was no significant difference between age groups.^3^ While positive blood culture is the gold standard for BSI diagnosis, it has a long turnaround time and potential false‐positive results caused by contamination.^4^ In addition, obtaining a qualified blood culture from pediatric patients, particularly neonates, can be challenging due to their low total blood volume.^5^, ^6^ Therefore, there is an urgent need to explore new diagnostic biomarkers that can quickly and easily predict the occurrence of BSI and sepsis in pediatric patients.

The immune response and inflammatory reaction are closely associated with hemocyte parameters such as white blood cell differential count, platelet count, and platelet‐associated parameters. Among these, the white blood cell differential count, including the neutrophil count and the lymphocyte count, are the oldest and most easily accessible inflammatory biomarkers for neonatal sepsis.^7^ Platelet (PLT) is a crucial component in thrombosis and hemostasis and also plays an essential role in immunomodulatory and inflammatory responses by interacting with immune cells and stimulating the release of cytokines.^8^, ^9^, ^10^, ^11^ Therefore, PLT and platelet parameters, such as platelet distribution width (PDW), large platelet ratio (LPCR), and especially mean platelet volume (MPV), have been explored as potential indicators of the occurrence of the inflammatory response.^12^, ^13^ However, leukocytes and PLT are easily affected by various physiological and pathological conditions of newborns, which limits their sensitivity and specificity in diagnosing neonatal sepsis.^14^, ^15^, ^16^, ^17^ Procalcitonin (PCT) is another inflammatory marker commonly used in sepsis, which is produced by thyroid C cells in normal status.^18^ Under inflammatory conditions, PCT is produced by extrathyroidal tissue in great quantities, making it a biomarker for sepsis.^19^, ^20^, ^21^ However, the neonatal PCT level exhibits physiological changes at 24–48 postnatal hours, and the PCT level physiological characteristics of preterm infants differ from those of normal term infants,^22^, ^23^, ^24^, ^25^ making it challenging to diagnose sepsis only by PCT values for neonates with different birth conditions. Early prediction and accurate diagnosis of BSI and sepsis in neonates can help reduce the inappropriate use of antibiotics and decrease the mortality of rate. However, current diagnostic methods, such as procalcitonin (PCT) or traditional inflammatory indicators like neutrophil count, lymphocyte count, platelet count, and platelet‐associated parameters, have limited predictive ability, resulting in low sensitivity and specificity in neonatal sepsis diagnosis.^17^, ^26^, ^27^, ^28^, ^29^ To address this issue, we investigated whether the combination of PCT and hemocyte parameters could improve diagnostic sensitivity and specificity for neonatal BSI and sepsis, compared to individual detection. Additionally, we explored the role of hemocytes in the inflammatory response of children with sepsis by analyzing the relative signaling pathways and hub genes in the Gene Expression Omnibus (GEO) database. Notably, for the first time, SPI1‐TYROBP‐FCER1G co‐expression has been found to contribute to the pathophysiology of pediatric sepsis through influencing the activity of neutrophils and platelets. The hub genes obtained during this GEO analysis may provide new targets for predicting sepsis in children.

## 2 MATERIALS AND METHODS

### 2.1 Research subjects

This retrospective study was conducted at the department of clinical laboratory of Children's Hospital of Chongqing Medical University between March 2020 and March 2022. We abstracted data for neonatal patients who received blood culture and other laboratory tests, including routine blood tests and procalcitonin (PCT) simultaneously, before initiating antibiotic therapy. The neonatal bloodstream infection (BSI) group included 262 cases of hospitalized neonates who underwent positive blood cultures. 157 neonates with negative blood cultures and diagnosed with noninfectious conditions, such as jaundice, were classified into the control group.

The inclusion criteria of this study were as follows: (1) infants with 28 days of postnatal age or less; (2) complete medical records and laboratory parameters; and (3) two sets of blood cultures should have consistent results. The exclusion criteria were as follows: (1) the postnatal age of infants as 28 days; (2) neonatal patients with previous diagnoses such as blood disorders, tumors, and autoimmune diseases; (3) two sets of blood cultures returned inconsistent results; and (4) neonatal patients with culture results of possible contamination such as isolation and cultivation of coagulase‐negative *staphylococcus* (CNS) from only one of two blood bottles or blood culture results of CNS but with a low level of PCT (<0.5 ng/ml).

This research was approved by the Institutional Review Board of Children's Hospital of Chongqing Medical University, and all data were collected anonymously which did not require informed consent of infants' parents.

### 2.2 Data collection

We collected data including age at onset, gender, nationality, area, multiple gestations (twins and triplets), gestational age, length of stay, weight at onset, Apgar score, blood culture results, time to positive, source of infection, results of routine blood tests, and PCT. The neutrophil to lymphocyte ratio (NLR), platelet to lymphocyte ratio (PLR), platelet to neutrophil ratio (PNR), mean platelet volume (MPV) to platelet count ratio (MPV/PLT), platelet count to PCT ratio (PLT/PCT), MPV to PCT ratio (MPV/PCT), red cell distribution width (RDW) to PCT ratio (RDW/PCT), RDW to platelet distribution width (PDW) ratio (RDW/PDW), RDW to platelet count ratio (RDW/PLT), platelet distribution width to platelet count ratio (PDW/PLT), and large platelet ratio (LPCR) to platelet count ratio (LPCR/PLT) were calculated based on complete blood count parameters.

### 2.3 Laboratory workup

2–3 ml of venous blood samples from patients of the BSI and the control groups were drawn into tubes with ethylenediaminetetraacetic acid (EDTA) and separation gel tubes, respectively. The EDTA samples were analyzed for complete blood count (CBC) using a Sysmex XN‐3000 automatic cell counter (Sysmex, Japan). Separation gel tubes were centrifuged at 4000g for 8 min in room temperature to obtain the serum. Serum samples were used for PCT using the Roche Cobas e602 analyzer (Roche, Switzerland). More than 2 ml blood was collected separately into two sets of blood culture bottles and incubated into BACTEC 9120 continuous‐monitory blood culture system (BD, USA). Bacterial identification and antibiotic susceptibility of positive blood culture samples were performed using VITEK 2 COMPACT (BioMerieux, USA).

### 2.4 Gene expression datasets

We obtained the validation datasets (GSE13904, GSE26378, and GSE26440) from the Gene Expression Omnibus (GEO) database, which had been normalized using robust multiple‐array average algorithm. The data of GSE13904, GSE26378, and GSE26440 were based on the GPL570 platform (Affymetrix Human Genome U133 Plus 2.0 Array).

### 2.5 Identification of differentially expressed genes

Differentially expressed genes (DEGs) between the sepsis group and the control group were identified using NetworkAnalyst (https://www.networkanalyst.ca/).^30^, ^31^ DEGs were defined as genes showing a 1.5‐fold change and an adjusted *p*‐value <0.05. The heat map and volcano plots of the DEGs were drawn using the pheatmap and the ggplot2 R package.

### 2.6 Gene ontology (GO) and pathway enrichment analyses

Enrichment analysis of DEGs was conducted by the Database for Annotation, Visualization, and Integrated Discovery (DAVID, https://david‐d.ncifcrf.gov/)^32^ to identify GO and REACTOME pathway enrichment. The GO term analysis included biological processes (BP), molecular functions (MF), and cellular components (CC). The bubble charts were drawn by the ggplot2 R package.

### 2.7 Gene set enrichment analysis (GSEA)

To determine the signaling pathway and biological process differences between the pediatric sepsis and control groups, we used the clusterProfiler R package to perform GSEA enrichment analysis.^33^, ^34^ The statistical significance was defined as a False Discovery Rate (FDR) < 0.25 and an adjusted *p*‐value <0.05.

### 2.8 Protein–protein interaction (PPI) network construction and hub genes

The PPI network was constructed with the STRING database (http://string‐db.org)^35^ and Cytoscape software.^36^ We identified the most significant module and hub genes by Molecular Complex Detection (MCODE) of Cytoscape.^37^

### 2.9 Correlation analysis

To explore the relationship between SPI1, FCER1G, and TYROBP, we used the ggplot2 R package to perform Spearman correlation analysis. *p* < 0.05 was considered statistically significant.

### 2.10 Statistical analysis

The statistical analysis was performed using SPSS version 26.0 (SPSS, USA). The normal distribution of the data was determined using the Shapiro–Wilk test. The descriptive statistics of continuous variables were expressed as the median and the interquartile range, and categorical variables were presented using frequency and percentage. The statistical difference of continuous variables between two groups were compared by the Mann–Whitney *U* test. The chi‐square test and the Fisher exact test were used to compare the categorical variables of the two groups. To assess the relationship between blood cell parameters and bloodstream infections in infants, we performed univariable and multivariable conditional logistic regression. Receiver operating characteristic (ROC) curve analysis was constructed to estimate the specificity and sensitivity of blood cell parameters and hub genes for predicting pediatric bloodstream infections and sepsis. Statistical significance was defined as *p* < 0.05 for all analyses.

## 3 RESULTS

### 3.1 Neonatal patient characteristics

In this research, 262 newborns with positive blood culture results were included in the BSI group and 157 neonates with negative results were included in the control group. As shown in Table 1, the median age at onset of the BSI group was 14 days, whereas the median age at onset of the control group was 2.13 days. We included age as a covariate for further analysis (Table 2). In comparison with the control group, the neonatal patients in the BSI group had longer length of stay, less gestational age, and weight at onset (Table 1), which means low gestational age and low weight of infants may be more susceptible to bloodstream infection. Meanwhile, the proportion of multiple gestations in the BSI group (27.1%) was significantly higher than that in the control group (9.6%) (Table 1). There was no significant difference in gender, nationality, and Apgar score between the two groups (Table 1).

**TABLE 1:** Demographic data of neonatal patients.

Variables	BSI (*n* = 262)	Control group (*n* = 157)	*p*‐value
Age at onset (days)	14 (7, 20)	2.13 (0.21, 12)	<0.001
Gender, male, *n* (%)	146 (55.7)	87 (55.4)	0.951
Nationality, Han, *n* (%)	246 (93.9)	146 (93)	0.717
Area, Chongqing, *n* (%)	227 (86.6)	117 (74.5)	0.002
Multiple gestation (*n*, %)	71 (27.1)	15 (9.6)	<0.001^a^
Gestational age (wk)	35 (32.57, 38.43)	38 (35.02, 39.57)	<0.001
Length of stay (days)	26 (16, 40)	14 (8, 23)	<0.001
Weight at onset (g)	2220 (1700, 3112.5)	3070 (2310, 3500)	<0.001
Apgar score			
1 min < 7, n/N (%)	33/197 (16.75)	16/101 (15.84)	0.841
5 min < 7, n/N (%)	6/194 (3.09)	6/95 (6.32)	0.197

^a^ Means the Fisher exact test.

**TABLE 2:** Laboratory characteristics of the BSI patients and control patients.

Variables	BSI (*n* = 262)	Control group (*n* = 157)	*p*‐value
PCT (ng/ml)	1.82 (0.33, 15.2)	0.3 (0.15, 0.935)	<0.001
WBC (×10^9^/L)	10.57 (6.37, 14.89)	12.83 (9.28, 17.67)	<0.001
LY (%)	40 (30, 50)	20 (10, 20)	<0.001
LY# (×10^9^/L)	3.69 (2.62, 4.90)	2.47 (1.49, 4.01)	<0.001
NE (%)	60 (50, 80)	60 (50, 70)	0.130
NE# (×10^9^/L)	6.12 (3.47, 10.22)	7.44 (4.55, 11.31)	0.021
NLR	2.39 (1.28, 5.22)	2.14 (1.12, 3.73)	0.049
PLT (×10^9^/L)	212 (131.75, 298)	285 (216.5, 363.5)	<0.001
MPV (fL)	11.6 (10.6, 12.4)	10.6 (9.9, 11.4)	<0.001
PDW (fL)	14.4 (12.3, 16.9)	11.8 (10.8, 13.55)	<0.001
LPCR (%)	36.55 (29.5, 42.5)	28.3 (24.45, 35.35)	<0.001
RDW (%)	16 (15.2, 17.3)	15.7 (15.15, 16.5)	0.043
PLR	82.77 (42.77, 157.09)	78.66 (59.74, 108.63)	0.125
PNR	35.93 (18.31, 66.22)	38.02 (21.39, 70.54)	0.181
MPV/PLT	0.055 (0.038, 0.092)	0.037 (0.028, 0.048)	<0.001
PLT/PCT	91.71 (14.31, 743.56)	863.27 (258.92, 2118.18)	<0.001
MPV/PCT	6.24 (0.755, 33.62)	34.85 (11.81, 77.57)	<0.001
RDW/PCT	8.71 (1.051, 51.32)	53.24 (17.37, 113.92)	<0.001
RDW/PDW	1.15 (0.98, 1.33)	1.34 (1.14, 1.48)	<0.001
RDW/PLT	0.076 (0.053, 0.126)	0.056 (0.043, 0.072)	<0.001
PDW/PLT	0.068 (0.045, 0.120)	0.042 (0.032, 0.058)	<0.001
LPCR/PLT	0.172 (0.109, 0.296)	0.099 (0.075, 0.147)	<0.001

Abbreviations: LPCR, large platelet ratio; LPCR/PLT, large platelet ratio to platelet count ratio; LY#, the count of lymphocyte; LY%, percentage of lymphocyte; MPV, platelet volume; MPV/PCT, mean platelet volume to PCT ratio; MPV/PLT, mean platelet volume to platelet count ratio; NE%, percentage of neutrophil; NE#, the count of neutrophil; NLR, the neutrophil to lymphocyte ratio; PCT, procalcitonin; PDW, platelet distribution width; PDW/PLT, platelet distribution width to platelet count ratio; PLR, platelet to lymphocyte ratio; PLT, platelet; PLT/PCT, platelet count to PCT ratio; PNR, platelet to neutrophil ratio; RDW, red cell distribution width; RDW/PCT, red cell distribution width to PCT ratio; RDW/PDW, red cell distribution width to platelet distribution width ratio; RDW/PLT, red cell distribution width to platelet count ratio; WBC, white blood cells.

To learn more about the demographic and clinical characteristics of the BSI group, we analyzed relevant indicators and the results are shown in Supplementary Table 1. Out of the 262 positive blood cultures, Gram‐positive constituted 52.3% (*n* = 137) of isolated organisms and the remainder was composed of Gram‐negative bacilli (*n* = 111, 42.3%) and fungi (*n* = 14, 5.4%). Depending on the time of symptoms onset, neonatal sepsis is divided into two types including early‐onset sepsis (EOS) (sepsis develops within 72 h after birth) and late‐onset sepsis (LOS) (sepsis occurs after 72 h after birth).^38^ To explore the relative proportions of early neonatal bloodstream infection due to Gram‐positive or Gram‐negative bacteria among infants, whose birth time was less than 72 h, we counted the distribution of bacteria, and the results showed that there was no significant difference in the proportion of the two types of bacteria. As shown in Supplementary Table 1, compared with the Gram‐positive group there were more newborns in the Gram‐negative group with Apgar scoring less than 7 at 1 min. What is more, in our study, Gram‐negative bacilli take longer culture time than positive than Gram‐positive cocci. Comparison of gender, nationality, area, multiple gestations, gestational age, length of stay, and weight at onset did not show significant difference among the Gram‐positive and Gram‐negative groups. Meanwhile, the source of infection in these two groups was predominantly the respiratory system (67.2% and 53.2%). S. epidermidis (77/137, 56.2%) was the most common category of Gram‐positive cocci for neonatal bloodstream infections. Notably, 19 of 85 (22%) *S*. *aureus*/epidermidis had been detected with methicillin resistance. The two most common pathogens isolated from the group with Gram‐negative bacilli were Klebsiella pneumoniae (44/111, 39.6%) and Escherichia coli (40/111, 36%). Multidrug resistance was detected in 21 Klebsiella pneumoniae isolates (data not shown).

### 3.2 Comparison of laboratory characteristics between the BSI group and the control group

Comparison of laboratory characteristics between the BSI group and the control group is summarized in Table 3. The results of procalcitonin (PCT) in the BSI group was significantly higher compared with the control group. A comparison of leukocyte classification and count showed that white blood cell (WBC) and neutrophil counts in the BSI group were significantly lower than the control group, whereas the count and the percentage of lymphocytes and the neutrophil to lymphocyte ratio (NLR) were significantly higher than that in the control group. In terms of laboratory results, the platelet‐related parameters such as platelet volume (MPV), platelet distribution width (PDW), large platelet ratio (LPCR), MPV/PLT, PDW/PLT, and LPCR/PLT in the BSI group showed a significant increase. By contrast, the PLT count, PLT/PCT, and MPV/PCT were significantly lower in the BSI group than in the control group. On comparing the red cell indices in the BSI group and the control group, it was found that the red cell distribution width (RDW) and RDW/PLT were significantly higher in the BSI group, whereas RDW/PCT and RDW/PDW were significantly lower in the BSI group. The BSI group and the control group indicated no significant differences in percentage of neutrophils, platelet to lymphocyte ratio (PLR), and platelet to neutrophil ratio (PNR).

**TABLE 3:** Univariate and multivariate analysis of variables associated with BSI.

Variables	Univariate		Multivariate			
					Adjusted^a^	
	OR (95%CI)	*p*‐value	OR (95%CI)	*p* Value	OR (95%CI)	*p*‐value
WBC (×10^9^/L)	0.963 (0.94–0.988)	0.003	‐	‐	‐	‐
LY (%)	1.164 (1.13–1.199)	<0.001	1.278 (1.208–1.352)	<0.001	1.301 (1.220–1.387)	<0.001
NE (%)	1.01 (0.998–1.021)	0.092	1.076 (1.039–1.114)	<0.001	1.079 (1.040–1.119)	<0.001
MPV (fL)	1.882 (1.548–2.289)	<0.001	‐	‐	‐	‐
PDW (fL)	1.329 (1.222–1.445)	<0.001	1.394 (1.209–1.608)	<0.001	1.284 (1.113–1.482)	0.001
LPCR (%)	1.091 (1.063–1.120)	<0.001	‐	‐	‐	‐
PLR	1.003 (1.001–1.006)	0.012	1.009 (1.004–1.014)	<0.001	1.009 (1.004–1.015)	0.001
PLT (×10^9^/L)						
≥320	Reference					
100–320	1.962 (1.260–3.054)	0.003	‐	‐	‐	‐
< 100	15.869 (4.662–54.015)	<0.001	‐	‐	‐	‐
PCT (ng/ml)						
< 0.5	Reference					
0.5–2	1.414 (0.834–2.4)	0.199	1.567 (0.581–4.228)	0.375	1.644 (0.573–4.722)	0.355
2–10	2.679 (1.492–4.808)	0.001	3.174 (1.075–9.37)	0.036	4.080 (1.260–13.219)	0.019
≥10	45 (10.745–188.456)	<0.001	67.851 (12.101–380.454)	<0.001	71.826 (11.852–435.292)	<0.001

*Note*: PLT ≥320 × 10^9^/L and PCT <0.5 ng/ml were sets of dummy variables.

Abbreviations: CI, confidence interval; OR, odds ratio.

^a^ Adjusted for age, length of stay, weight at onset, gestational age, and time to positive.

### 3.3 Multivariate analysis of variables associated with neonatal bloodstream infections

To identify the factors that could discriminate between positive blood culture and negative blood culture in neonatal patients, a binary logistic regression analysis was performed (Table 3). Firstly, a univariate binary logistic regression analysis was performed. According to the reference range, PLT ≥320 × 10^9^/L and PCT <0.5 ng/ml were set as dummy variables. Secondly, the variables with a *p*‐value <0.2 in the univariate binary logistic regression analysis were entered into multivariable stepwise logistic regression analysis, in order to investigate the factors independently associated with neonatal BSI. The results of the binary logistic regression analysis showed that the percentage of lymphocyte (adjusted odds ratio (AOR): 1.301, 95% CI: 1.220–1.387, and *p* < 0.001) and the percentage of neutrophil (AOR: 1.079, 95% CI:1.040–1.119, and *p* < 0.001), PDW(AOR: 1.284, 95% CI:1.113–1.482, and *p* = 0.001), PLR (AOR: 1.009, 95% CI: 1.004–1.015, and *p* = 0.001), and PCT (2 ng/ml ≤ PCT <10 ng/ml, AOR: 4.080, 95% CI: 1.260–13.219, *p* = 0.019; PCT ≥10 ng/ml, OR: 71.826, 95% CI: 11.852–435.292, and *p* < 0.001) were the independent predictors of neonatal BSI. The predictive equation for neonatal BSI was (P) = −16.524 + 0.245×LY%+0.073×NE%+0.332×PDW+0.009×PLR +1.55×PCT (2–10 ng/ml) +4.217×PCT (≥10 ng/ml).

The diagnostic performance of related indicators and the model were compared by the receiver operating characteristic (ROC) curve.

**FIGURE 1:**
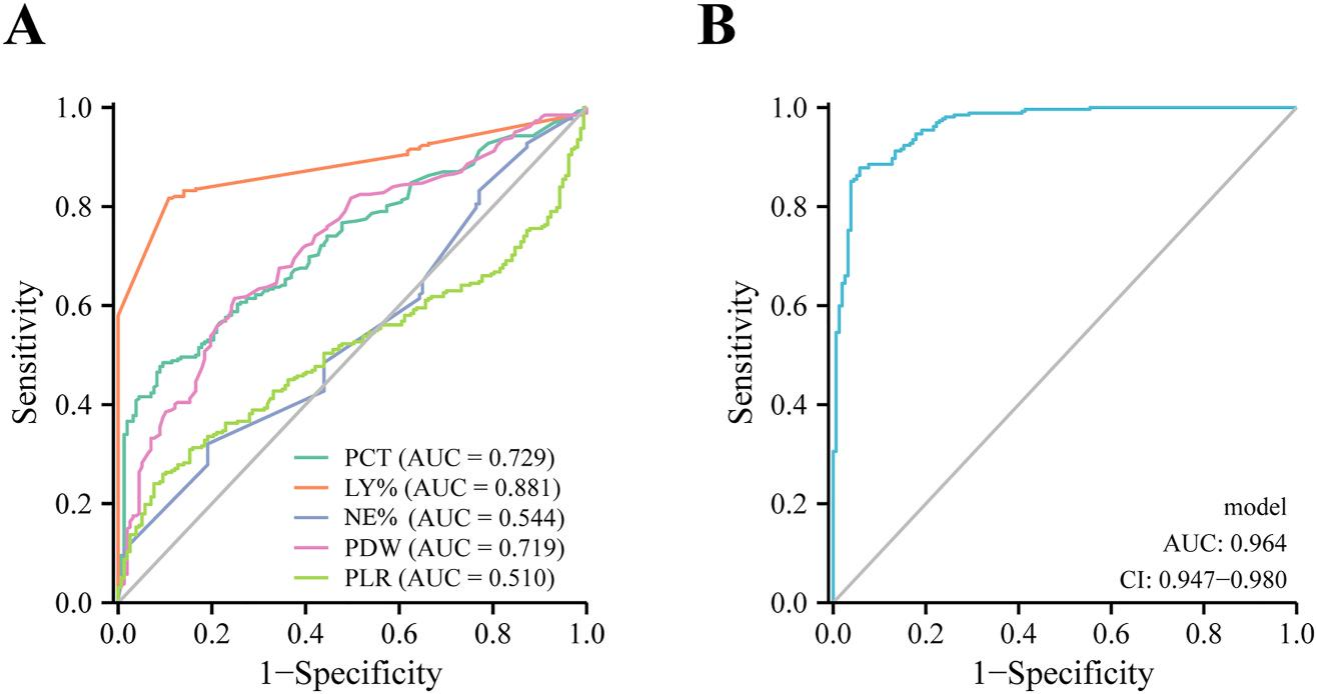
The ROC curve of related indicators and the model for differential diagnosis of BSI. (A) The ROC curve of PCT, LY%, NE% PDW, and PLR. (B) The ROC curve of the model. PCT: procalcitonin, PDW: platelet distribution width, and PLR: platelet to lymphocyte ratio.

To further investigate the diagnostic performance of individual and joint detection of these indicators, and the diagnostic value of the predictive model, we performed a receiver operating characteristic (ROC) curve and calculated the area under the curve (AUC), the optimal cutoff value, sensitivity, specificity, positive predictive value (PPV), and negative predictive value (NPV). As shown in Figure 1 and Table 4, PCT, LY%, NE%, PDW, and PLR demonstrated a moderate ability to diagnose neonatal patients with bloodstream infections (BSI). The model, which combined PCT, LY%, NE%, PDW, and PLR, had the highest AUC (0.968), sensitivity (92%), specificity (87.3%), PPV (92%), and NPV (86.7%) in ROC analysis, which means this prediction model had better diagnostic efficacy for neonatal BSI.

**TABLE 4:** The diagnostic performance of related indicators and the model were compared by the receiver operating characteristic (ROC) curve.

Parameter	AUC	95% CI	Cutoff value	Sensitivity (%)	Specificity (%)	PPV (%)	NPV (%)
PCT (ng/ml)	0.729	0.681–0.776	2.53	66.4	63.1	75	52.9
LY (%)	0.881	0.849–0.913	29.6	88	72	74	70
NE (%)	0.544	0.488–0.599	70.15	32	80	73	42
PDW (fL)	0.719	0.669–0.768	13.55	82.4	48.4	72.7	62.3
PLR	0.510	0.456–0.565	166.15	24	92	84	42
Model	0.968	0.954–0.983	0.675	92	87.3	92	86.7

### 3.4 Differentially expressed genes (DEGs) between sepsis patients and healthy control patients

Sepsis is based on pathogenic bacteria invasion of the bloodstream (bloodstream infection, BSI) or the release of microbial metabolites and products into the bloodstream, which leads to the activation of the immune system and the promotion of systemic inflammatory reaction.^39^ Thus, further research is needed to explore the immunological changes during sepsis containing the activation markers and pathways of neutrophils, lymphocytes, and platelet to find better early septic biomarkers. We obtained 50 children from GEO databases (GSE13904), with 32 sepsis patients and 18 healthy control patients. Blood samples were collected on the day when all patients were divided into the sepsis group and the control group. To explore the underlying mechanism between sepsis and parameters of blood cells in children, we analyzed the differential gene expression between the two groups by NetworkAnalyst. As shown in Supplementary Figure 1A, a heat map was constructed by |log FC| > 1.5 and an adjusted *p*‐value <0.05 and indicated the top 50 differentially expressed genes (DEGs), and a volcano plot displayed all DEGs between the groups (Supplementary Figure 1B). Additionally, a total of 261 DEGs (|log FC| > 1.5 and adjusted *p*‐value <0.05), including 140 upregulated DEGs and 101 downregulated DEGs, were selected for further analysis to explore the correlation with the predictive value of sepsis in children.

### 3.5 GO, REACTOME, and GSEA enrichment analysis of DEGs

**FIGURE 2:**
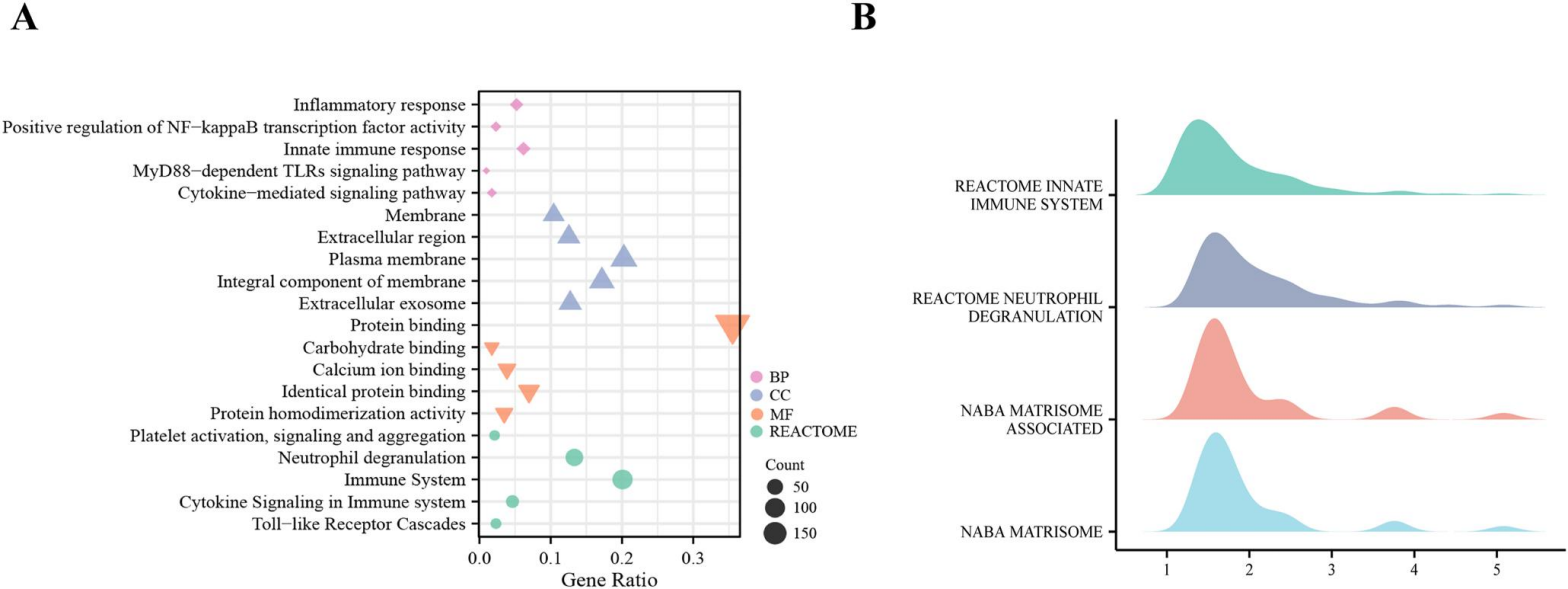
GO, REACTOME, and GSEA enrichment analysis of DEGs. (A) Gene Ontology (GO) and REACTOME enrichment analysis. The size of dots indicates the gene number. (B) GSEA analysis revealing the DEGs were significantly mainly in the innate immune system and the neutrophil degranulation pathway. BP: biological process; CC: cellular component; and MF: molecular function.

To explore the potential function of these DEGs, we performed gene ontology (GO) and REACTOME pathway enrichment analyses by DAVID. GO and REACTOME pathway enrichment analyses revealed that the two groups had significant differences (adjusted *p*‐value< 0.05) in 46 biological process (BP), 27 molecular function (MF), 40 cellular component (CC), and 45 REACTOME pathway terms. Biological process attributed to these DEGs included “inflammatory response”, “positive regulation of NF‐kappa B transcription factor activity”, “innate immune response”, “myD88‐dependent toll‐like receptor signaling pathway”, and “cytokine‐mediated signaling pathway” (Figure 2A). Cellular component terms mainly included “membrane”, “extracellular region”, “plasma membrane”, “integral component of membrane”, and “extracellular exosome” (Figure 2A). Primary terms within molecular function included “protein binding”, “carbohydrate binding”, “calcium ion binding”, “identical protein binding”, and “protein homodimerization activity” (Figure 2A). Furthermore, the REACTOME pathway analysis revealed that the DEGs were concentrated in “platelet activation, signaling and aggregation”, “neutrophil degranulation”, “immune system”, “cytokine signaling in immune system”, and “toll‐like receptor cascades” (Figure 2A). Subsequent gene set enrichment analysis (GSEA) showed that the pediatric sepsis group was mainly involved in the innate immune system and the neutrophil degranulation pathway (Figure 2B). The results indicated that these DEGs were related to blood cells and played an important role in the inflammatory response caused by sepsis, which merits further investigation.

### 3.6 Protein–protein interaction (PPI) analysis of DEGs and evaluation of the diagnostic performance of hub genes by ROC curve

**FIGURE 3:**
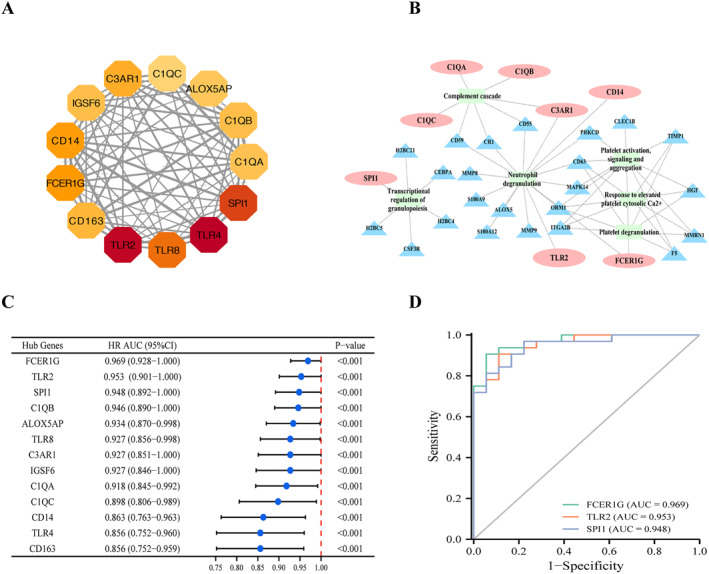
Protein–protein interaction (PPI) analysis of DEGs and ROC curve analysis of hub genes. (A) One significant module was identified based on the degree of importance, which contained 13 hub genes. The color of the node reflects its connectivity degree score. (B) The network showed mostly hub genes were related to the pathway of activation of neutrophils, platelet, and complement system. (C) The forest map of the AUCs of all the hub genes. (D) The ROC curve of FCER1G, TLR2, and SPI1 in the value of predicting diagnosis.

We next performed a protein–protein interaction (PPI) network to explore the interplay between these DEGs. PPI network was constructed with the STRING database and Cytoscape software, which contained 198 nodes and 671 edges. The Cytotype MCODE was applied in the selection of a significant module. As Figure 3A shows, the significant module included 13 nodes and 71 edges and contained 13 hub genes: TLR2, TLR4, TLR8, SPI1, FCER1G, CD14, C3AR1, CD163, IGSF6, C1QA, C1QB, C1QC, and ALOX5AP. Notably, mostly hub genes were related to the activation of neutrophils, platelet, and complement system, which indicated that the blood cells play an important role in diagnostic pediatric sepsis (Figure 3B). This result was consistent with the conclusions based on our previous clinical data analysis. To further confirm the diagnostic performance of the 13 hub genes for sepsis in children, we constructed a ROC curve analysis. As shown in Figure 3C, the AUCs of all the hub genes exceeded 0.8, and it was indicated that these hub genes had a highly diagnostic significance for pediatric sepsis. Furthermore, FCER1G, TLR2, and SPI1 were the top 3 AUCs (AUCs = 0.969, 0.953, and 0.948, respectively) (Figure 3D). Recent studies have shown that SPI1 and FCER1G were closely related to the immune infiltration and prognosis of several types of tumors.^40^, ^41^ Additionally, SPI, TYROBP, and FCER1G formed a conserved immune network that influenced the oncogenesis and prognosis of osteosarcoma^42^; however, their levels and functions in sepsis were not clear. Compared with normal children, pediatric patients with sepsis (GSE13904) or septic shock (GSE26378, GSE26440) had significantly higher expression levels of SPI1, TYROBP, and FCER1G (Supplementary Figure 2A‐C). Furthermore, the expressional levels of SPI1 and FCER1G were positively correlated with TYROBP (Supplementary Figure 2D‐F). Taken together, PPI and enrichment analysis demonstrated that the DEGs and hub genes were related to the activation of blood cells, which indicated that the activated blood cells play an important role in diagnostic pediatric sepsis.

## 4 DISCUSSION

Bloodstream infection (BSI) is a serious systemic inflammation that affects the morbidity and mortality of neonatal intensive care units (NICUs).^43^ At birth, mucosal surfaces of neonates are devoid of protective endogenous flora, and both function and quantity of neonatal innate immunity are not fully mature.^44^, ^45^, ^46^ Thus, compared with adults, neonates are more prone to BSI and have a high risk of developing sepsis, severe sepsis, septic shock, and even death.^47^ Blood culture, the gold standard for diagnosis of neonatal BSI and sepsis, is limited for early diagnosis by the long report time and the large blood volume.^48^, ^49^ In a clinic, circulating blood biomarkers are readily available and routinely measured, which can provide vital information for the early diagnosis of neonatal BSI and sepsis.

PCT is an acute‐phase reactant that can increase a thousandfold under bacterial infection, making it a useful biomarker for the diagnosis of adult sepsis.^50^ Furthermore, the high level of PCT correlates with severity of infection.^47^, ^51^ However, in neonatal sepsis, the clinical application of PCT has limitations. Firstly, the predictive value of PCT in neonatal sepsis during the early neonatal period is confounded by the physiological differences observed among preterm, late‐preterm, and term infants.^47^, ^52^, ^53^ Setting the cutoff of PCT values for newborns with different gestational ages may increase the diagnostic accuracy of neonatal sepsis, but recent research showed diverging opinions on the PCT cutoff values and there was no uniform conclusion.^54^, ^55^ Secondly, the heterogeneity in sample timing, cutoff values, patient population, and definition of early‐onset sepsis (EOS) or late‐onset sepsis (LOS) can influence the outcomes of PCT.^56^, ^57^ Lastly, in specific patient populations, such as some critically ill pediatric patients admitted to the pediatric intensive care unit (PICU), PCT was not reliable to differentiate bacterial infections from other nonbacterial infection diseases.^58^, ^59^ Therefore, PCT could not be used as a stand‐alone test for neonatal sepsis diagnosis.

Complete blood count parameters, least costly and readily available, is the earliest laboratory monitoring index for neonatal BSI and sepsis. Absolute lymphocyte count, absolute neutrophil count, and platelet count are commonly used for neonatal sepsis diagnosis.^60^, ^61^ As neutrophils are the dominant cells in microbial infections, they can be quickly depleted. Recent results showed that neutropenia could be one of the signs in the diagnosis of neonatal sepsis.^62^, ^63^ However, the neonatal neutrophil count is easily affected by other factors, such as postnatal age, meconium aspiration, and congenital neutropenia, which limited its single use in neonates with sepsis.^14^, ^15^, ^16^ Thrombocytopenia, a low platelet count, is an early and independent indicator of neonatal sepsis‐related death.^64^ Platelet‐related parameters such as platelet to lymphocyte ratio (PLR), MPV, PDW, and LPCR also suggest a role in diagnosing neonatal sepsis, but their specificity and sensitivity varies greatly among studies.^17^, ^29^, ^65^, ^66^ In this study, we found that combining the results of PCT, lymphocyte percentage, neutrophil percentage, and PLR greatly increased the specificity and sensitivity of diagnosing neonatal sepsis.

In order to understand how neutrophils, lymphocytes, and platelets are activated in the inflammatory response associated with pediatric sepsis at the molecular level, a bioinformatics analysis was conducted on gene differential expression, signaling pathways, and protein–protein interactions. Two top differentially expressed genes were identified in the PPI network analysis, FCER1G and SPI1, both of which are involved in a conserved immune‐related network. FCER1G, known as FcRγ, is an innate immune gene, which transduced activation signals from multiple immunoreceptors and is involved in mediating various immune responses.^67^, ^68^ As a key molecule in immune signaling pathways, FCER1G is functionally linked to the activation of neutrophils and is also concerned with the activation of platelets.^69^ Previous studies showed that FCER1G was related to the development of various diseases, including multiple myeloma, bleeding disorder, and mitochondrial complex I deficiency.^70^ Furthermore, FCER1G is the essential molecule involved in the progression of many kinds of tumors, such as clear cell renal cell carcinoma (ccRCC), meningioma, acute myeloid leukemia (AML), and childhood leukemia.^71^, ^72^, ^73^, ^74^

SPI1, as a transcription factor, can be regulated by FCER1G.^75^, ^76^ Notably, SPI1 (PU.1) is associated with the differentiation of various immune cells, including granulocytes, lymphocytes, monocytes, and dendritic cells.^77^, ^78^, ^79^, ^80^ In addition, our results showed that in pediatric sepsis, the expressional level of SPI1 and FCER1G were positively correlated with TYROBP (Supplementary Figure 2D‐F). TYROBP, also known as DAP12, mediates the transduction of activation signals to all kinds of immune cells, especially NK cells, whose activation depends on the DAP12 pathway.^81^ Besides, TYROBP is related to the immune infiltration of various immune cells, such as neutrophils, monocytes, and macrophages, and is involved in the regulation of the tumor microenvironment.^41^, ^82^, ^83^ The SPI1‐TYROBP‐FCER1G immune‐related network has been reported to affect the immune invasion and the prognosis of multiple tumors.^40^, ^41^, ^42^, ^71^, ^72^, ^73^, ^74^ However, the role of the SPI1‐TYROBP‐FCER1G network in pediatric sepsis has not been previously reported. Our study for the first time finds that this immune‐related network may contribute to the pathophysiology of pediatric sepsis through the influence of the activation of neutrophils and platelets. These results indicated that SPI1, FCER1G, and TYROBP may be potential septic biomarkers.

Additionally, CD14 and CD163 were also found to be part of the hub genes in the PPI network of hub genes. CD14 is an LPS receptor that is involved in TLR signaling and enhances the TLR‐mediated immune response.^84^, ^85^, ^86^, ^87^ Recent research showed that soluble CD14, known as presepsin, has diagnostic value for pediatric sepsis patients.^55^ However, the monocyte CD14 (mCD14) has limited diagnostic efficiency and a monitoring role for sepsis, and is therefore not suitable for use as a septic biomarker in NICUs.^88^ CD163 plays an anti‐inflammatory and antioxidant role in the inflammatory network.^89^ sCD163, the soluble situation of CD163, has been confirmed and can be used as a biomarker for early diagnosis of severe infection and sepsis in adults, and is one of the reference indicators for judging the immune status of children with sepsis.^90^, ^91^, ^92^ Our study provides a theoretical basis for CD14 and CD163 as new biomarkers for predicting sepsis in children.

This present work also has several limitations. It was a single‐center retrospective study; thus, the findings should be further verified with multicenter and large datasets. Additionally, since there are no available GEO datasets specifically related to neonatal sepsis, the conclusions drawn from analyzing the datasets of children's sepsis should be further confirmed by constructing a neonatal sepsis dataset. Moreover, the identification of hub genes and pathways was solely based on open databases, and therefore, further functional experiments are required for future research.

## 5 CONCLUSIONS

**FIGURE 4:**
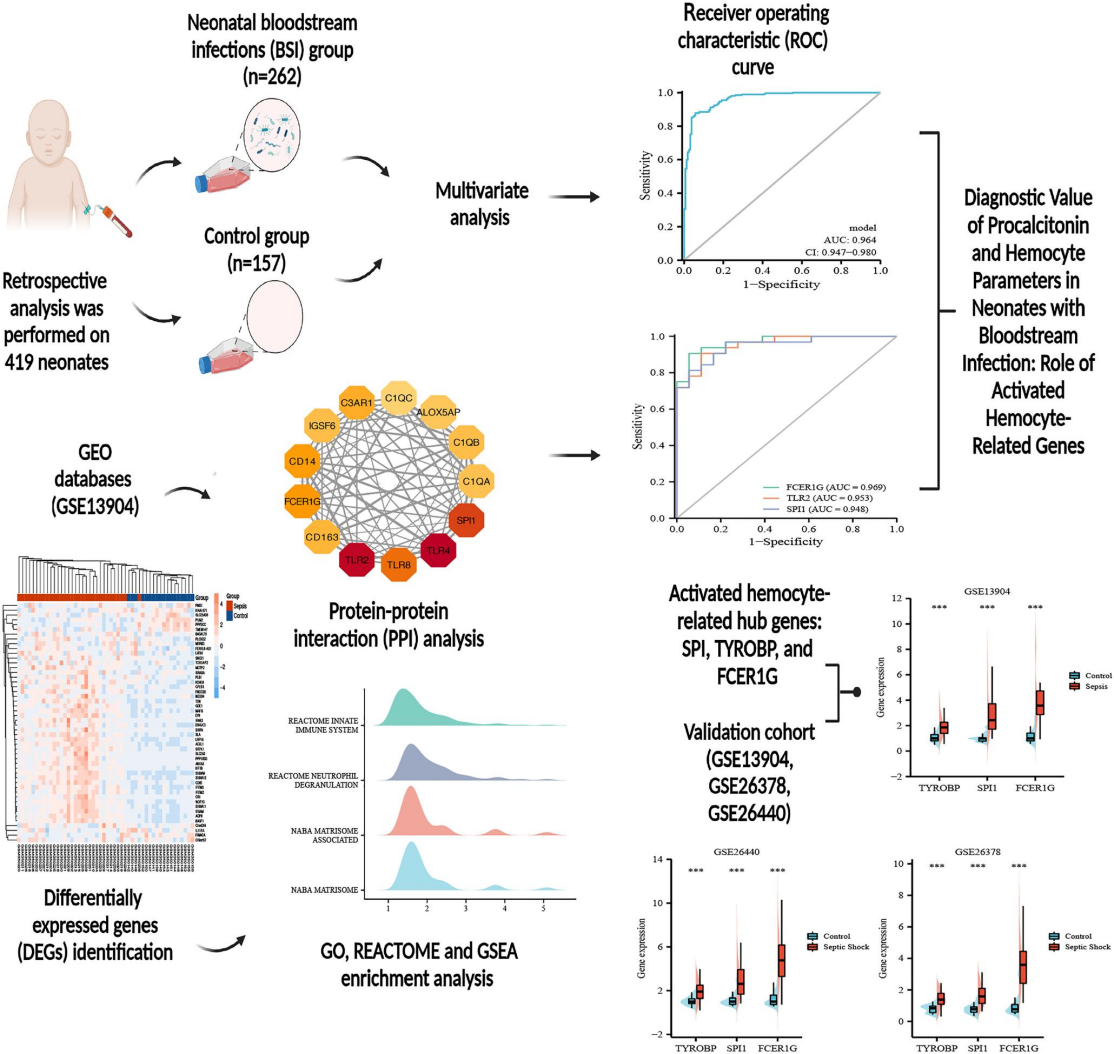
Flowchart of the study. Created with BioRender.com.

The model based on readily available biological indicators, consisting of PCT, LY%, NE%, PDW, and PLR, had better diagnostic efficacy for neonatal BSI. Furthermore, our study for the first time showed that the SPI‐TYROBP‐FCER1G co‐expression network plays an important role in pediatric sepsis immune response by affecting the activation of neutrophils and platelets, which may be new biomarkers of pediatric sepsis (Figure 4).

## AUTHOR CONTRIBUTIONS

YYT and NSH designed and performed the study. YYT, HDP, and NSH collected the clinical data. QL revised the manuscript. NSH wrote the manuscript and supervised the whole work. All authors read and approved the final manuscript. All authors agreed to the publication of this article.

## CONFLICT OF INTEREST STATEMENT

The authors declare no conflict of interest.

## ETHICS STATEMENT

This research was approved by the Institutional Review Board of Children's Hospital of Chongqing Medical University (ID: 2022‐211), and all methods were carried out in accordance with relevant guidelines and regulations.

## PATIENT CONSENT STATEMENT

The need for informed consent was waived by the Institutional Review Board of Children's Hospital of Chongqing Medical University.

## Supporting information

Supplementry material S1

Supplementry material S2

Supplementry material S3

## Data Availability

The publicly available datasets can be found here: https://www.ncbi.nlm.nih.gov/geo/query/acc.cgi (GEO datasets). The original contributions presented in the study are included in the article material; further inquiries can be directed to the corresponding author.
