# Supplementary material for: Diagnostic value of procalcitonin and hemocyte parameters in neonates with bloodstream infection: Role of activated hemocyte‐related genes
Source: Pediatr Discov. 2024 May 23;2(4):e56. doi: 10.1002/pdi3.56 (PMC12118178; doi:10.1002/pdi3.56)
Supplement: Supplementary file 1 — Supplementry material S1 [file PDI3-2-e56-s001.docx]

**SuppInfo_Table1** Demographic data of BSI patients with Gram-Positive or Gram-Negative

| **Varialbles** | **Gram-Positive**  **(n=137)** | **Gram-Negative**  **(n=111)** | **P value** |
| --- | --- | --- | --- |
| Early-onset sepsis, n (%) | 10 (7.3) | 15 (13.5) | 0.106 |
| Gender, male, n (%) | 74 (54) | 65 (58.6) | 0.473 |
| Nationality, Han, n (%) | 127 (92.7) | 107 (96.4) | 0.210 |
| Area, Chongqing, n (%) | 115 (83.9) | 99 (89.2) | 0.232 |
| Multiple gestation (n, %) | 29 (21.2) | 38 (34.2) | 0.054^†^ |
| Gestational age (wk) | 35.3 (33.17, 38.42) | 34.93 (32.29, 38.42) | 0.497 |
| Length of stay (days) | 29.55 (16.5, 38) | 31.09 (15, 44) | 0.734 |
| Weight at onset (g) | 2471.84 (1760, 3190) | 2337.56 (1698, 3110) | 0.19 |
| Apgar score |  |  |  |
| 1min < 7, n/N (%) | 9/94 (9.6) | 21/86(24.4) | **0.008** |
| 5 min < 7, n/N (%) | 2/94 (2.1) | 3/86 (3.5) | 0.671^a^ |
| Time to positive (h) | 18.12 (10.56, 22.09) | 18.51 (8.9, 17.9) | **0.009** |
| Source of infection, n (%) |  |  | 0.086 |
| Respiratory system | 92 (67.2) | 59 (53.2) |  |
| NEC | 8 (5.8) | 5 (4.5) |  |
| Multiple system | 22 (16.1) | 30 (27) |  |
| Others or unexplained sources | 15 (10.9) | 17 (15.3) |  |

Note: ^†^ means Fisher exact test. Early-onset sepsis means the birth time of infants with sepsis was less than 72 hours.

**Figure legends**

**SuppInfo_Fig 1**. **Identification of differentially expressed genes (DEGs).** (A) Heatmap of top 50 DEGs in the GSE13904 cohort. (B) Volcano map of DEGs in the GSE13904 cohort. Blue and red colors represent low and high relative gene expression levels, respectively.

**SuppInfo_Fig 2.** **Expression level of SPI1, TYROBP, and FCER1G in pediatric patients with sepsis or septic shock**. SPI1, TYROBP, and FCER1G expressed higher in pediatric sepsis (A), septic shock (B-C), and correlated significantly (D-F). ***P<0.001 vs. control group.
