# Supplementary figures and images for: Diagnostic value of procalcitonin and hemocyte parameters in neonates with bloodstream infection: Role of activated hemocyte‐related genes
Source: Pediatr Discov. 2024 May 23;2(4):e56. doi: 10.1002/pdi3.56 (PMC12118178; doi:10.1002/pdi3.56)

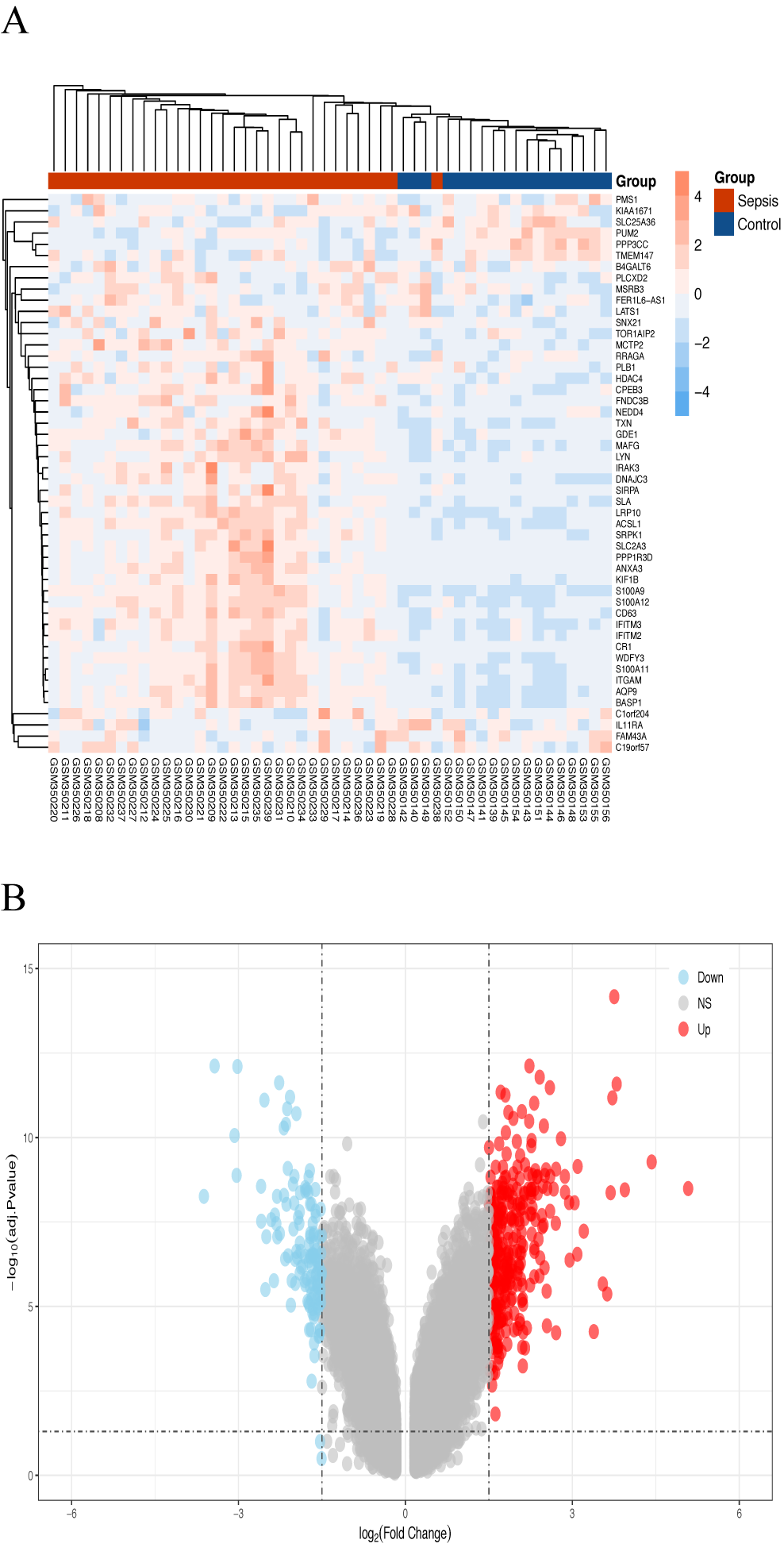

Supplement: Supplementary file 2 — Supplementry material S2 [file PDI3-2-e56-s003.tiff]

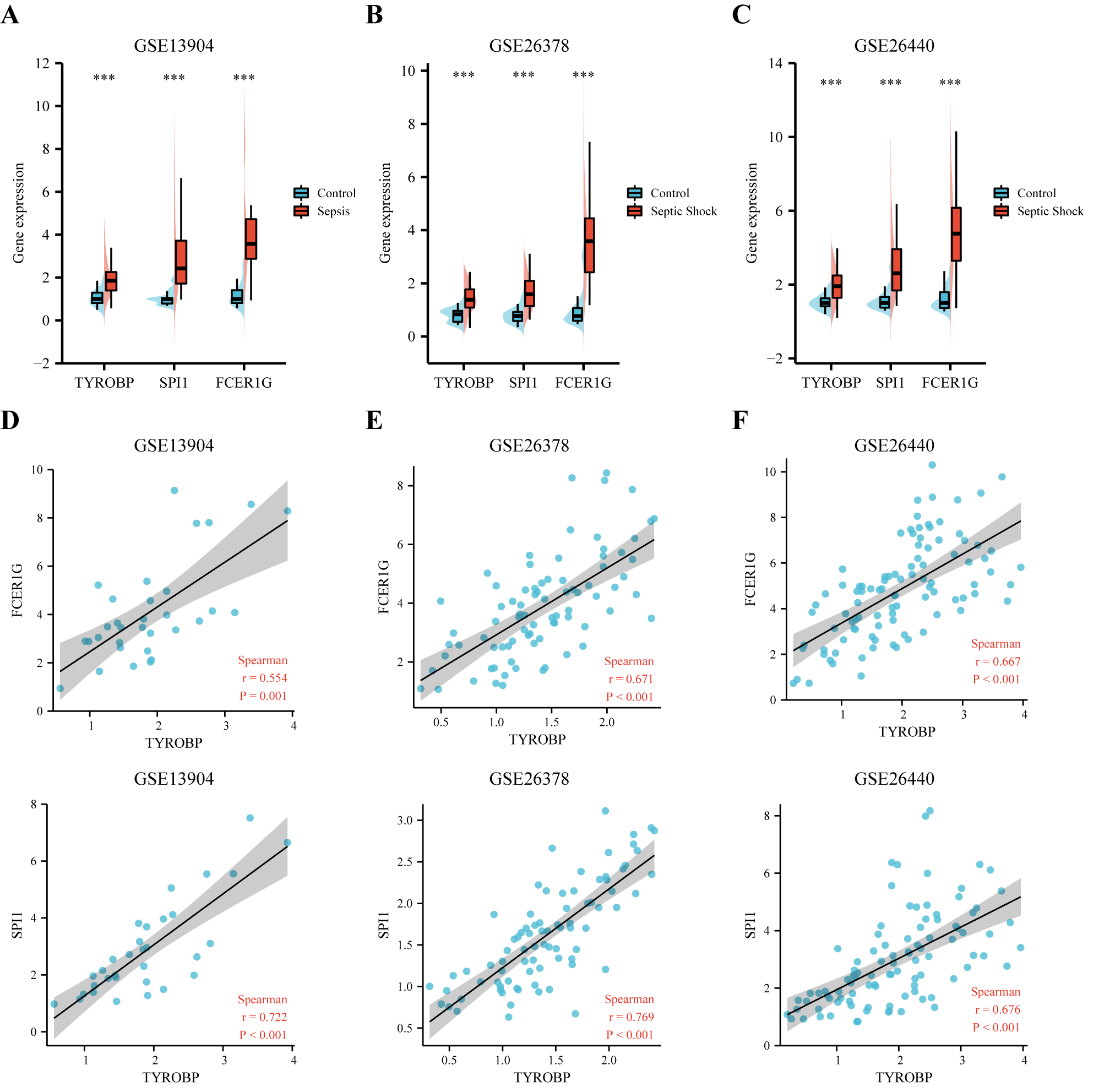

Supplement: Supplementary file 3 — Supplementry material S3 [file PDI3-2-e56-s002.tiff]
